# A stable and high-energy aqueous aluminum based battery†

**DOI:** 10.1039/d2sc03455g

**Published:** 2022-07-28

**Authors:** Renqian Tao, Caitian Gao, Erqing Xie, Bin Wang, Bingan Lu

**Affiliations:** Key Laboratory for Magnetism and Magnetic Materials of the Ministry of Education, School of Physical Science and Technology, Lanzhou University Lanzhou 730000 P. R. China; School of Physics and Electronics, State Key Laboratory of Advanced Design and Manufacturing for Vehicle Body, Hunan University Changsha 410082 P. R. China ctgao@hnu.edu.cn; School of Physics and Electronic Engineering, Xinxiang University Xinxiang 453000 P. R. China

## Abstract

Aqueous aluminum ion batteries (AAIBs) have received growing attention because of their low cost, safe operation, eco-friendliness, and high theoretical capacity. However, one of the biggest challenges for AAIBs is the poor reversibility due to the presence of an oxide layer and the accompanying hydrogen evolution reaction. Herein, we develop a strongly hydrolyzed/polymerized aluminum–iron hybrid electrolyte to improve the electrochemical behavior of AAIBs. On the one hand, the designed electrolyte enables aluminum ion intercalation/deintercalation on the cathode while stable deposition/stripping of aluminium occurs on the anode. On the other hand, the electrolyte contributes to the electrochemical energy storage through an iron redox reaction. These two reactions are parallel and coupled through an Fe–Al alloy on the anode, thus enhancing the reversibility and energy density of AAIBs. As a result, this hybrid-ion battery delivers a specific volumetric capacity of 35 A h L^−1^ at the current density of 1.0 mA cm^−2^, and remarkable stability with a capacity retention of 90% over 500 cycles. Furthermore, the hybrid-ion battery achieves a high energy density of approximately 42 W h L^−1^ with an average operating voltage of 1.1 V. This green electrolyte for high-energy AAIBs holds promises for large-scale energy storage applications.

## Abstract 2

A hybrid-ion aqueous aluminium ion battery (HIAAIB) with nickel hexacyanoferrate as the cathode, Al as the anode and a polymerized Al–Fe hybrid electrolyte is reported. During discharge, an Fe–Al alloy forms at the anode, improving performance by relieving corrosion.

## Graphical abstract

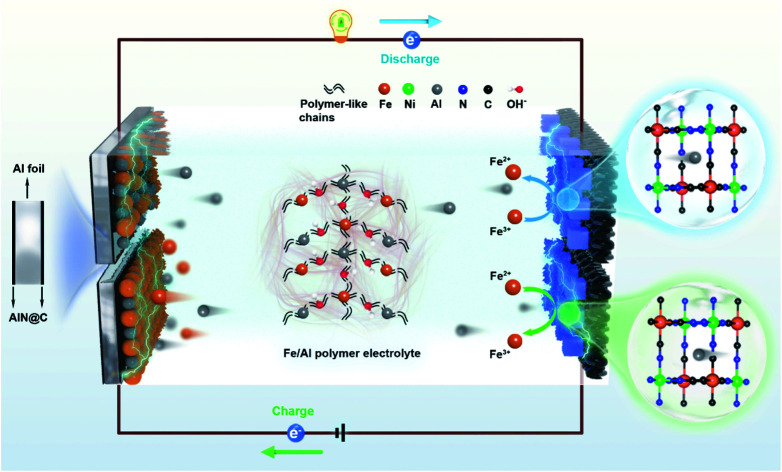

## Introduction

Owing to its low-cost, safety, and high theoretical capacity, rechargeable aqueous aluminum ion batteries (AAIBs) have attracted extensive attention.^1–8^ However, they still suffer from several critical issues, such as poor reversibility due to the limited deposition/stripping efficiency of aluminum ions and low energy density caused by the accompanying hydrogen evolution reaction.^9–14^ Comparing the solutions to these issues, strategies involving the tuning of the electrolyte are more significant, for instance using electrolyte additives to improve electrolyte ion transfer rate, applying eutectic electrolytes to broaden the voltage window, and mixing multivalent ions to compensate electrode consumption and improve the deposition/stripping efficiency.^1,15,16^ Among them, the hybrid-ion battery has many intriguing advantages based on its unique design, such as the simple preparation process, low cost, as well as large-scale applications,^17^ while it still faces the challenge of relatively low energy density and poor cycle performance.^9,18–20^ An Al–Zn ion battery with Al_2_(SO_4_)_3_/Zn(CHCOO)_2_ as a hybrid electrolyte was reported to have an energy density of 40 W h kg^−1^ after 200 cycles,^21^ but the cycle performance and capacity retention rates were not satisfactory. Similarly, Li *et al.* reported that a full aqueous aluminum ion battery composed of a vanadium potassium cathode and Al anode exhibited reversible charge/discharge behaviors in 0.5 M AlCl_3_@12 M LiTFSI electrolyte,^22^ while the battery cycling stability was only 64.6% after 300 cycles. Therefore, it is critical to pursue a hybrid-ion system to assist AAIBs with long cycle life and high energy density.^15,17,22–25^

**Fig. 1:**
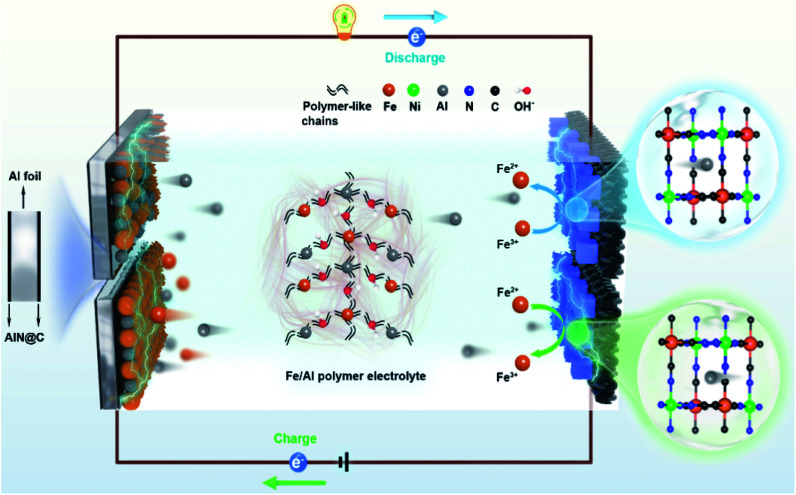
The structure and schematic illustration of a hybrid-ion aqueous aluminum ion battery (HIAAIB).

In this work, we demonstrate the enhancement of the energy density of AAIBs through the surface reaction of iron pairs in a newly developed electrolyte, *i.e.* a hybrid-ion aqueous aluminum ion battery (HIAAIB). Fig. 1 depicts the reaction scheme of the HIAAIB. Specifically, the battery is constructed with nickel hexacyanoferrate (NiFe-PBA) as the cathode, Al metal coated with AlN (c-Al) as the anode, and hydrolyzed/polymerized aluminum–iron hybrid electrolyte (poly-aluminum ferric chloride, PAFC) as the electrolyte. The PAFC electrolyte is generated by the polymerization reaction of aluminum chloride and iron chloride (see the details in Fig. S1†). During the discharging process, aluminum ions are intercalated into NiFe-PBA, and the ferric ions are reduced simultaneously on the surface of the cathode, while during the charging process, aluminum ions are deintercalated, and the oxidation of the ferrous ions occur on the surface of the cathode. At the same time, at the anode, deposition of ferrous ions and aluminum ions occurs, ultimately forming an Fe–Al alloy, which assists in capacity increase and cycle performance by relieving the anode corrosion. Impressively, the HIAAIB delivers a specific volumetric capacity of 35.0 A h L^−1^ with the current density of 1.0 mA cm^−2^, the capacity remains over 90% after 500 cycles. In addition, the hybrid-ion battery shows excellent rate capability; a specific volumetric capacity of ∼20 A h L^−1^ is achieved at a high current density of 5.0 mA cm^−2^. Moreover, the energy density of the HIAAIB is as high as 42 W h L^−1^, which is a great improvement compared to AAIBs.

## Results and discussion

**Fig. 2:**
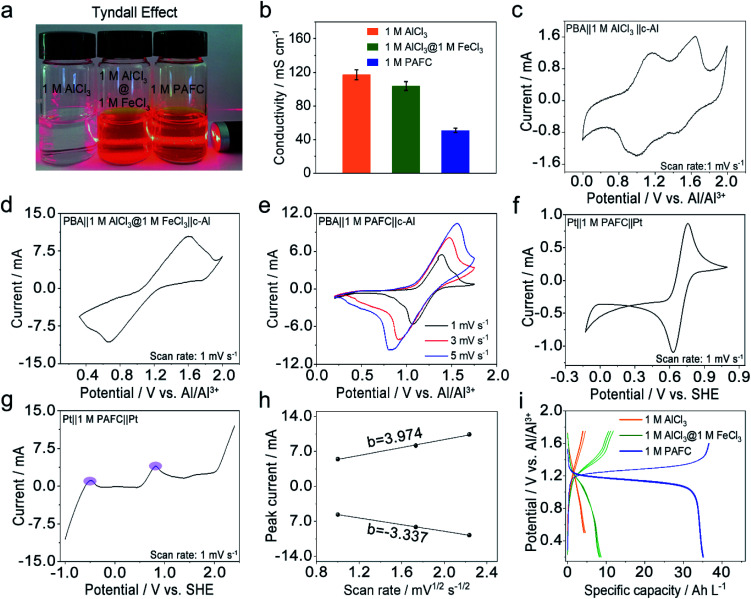
The basic physical and electrochemical properties of PAFC electrolyte. (a) Digital images and Tyndall effect of 1 M AlCl_3_, mixed 1 M AlCl_3_@FeCl_3_ and 1 M PAFC, respectively. (b) Ion conductivity of 1 M AlCl_3_, 1 M AlCl_3_@FeCl_3_ and 1 M PAFC, respectively. (c and d) Cyclic voltammetry (CV) curve of 1 M AlCl_3_ and 1 M AlCl_3_@1 M FeCl_3_ electrolyte at 1 mV s^−1^, the cathodes and anodes are NiFe-PBA and c-Al. (e) CV curves of NiFe-PBA‖1 M PAFC‖c-Al at 1, 3 and 5 mV s^−1^, respectively. (f) CV curve of Pt‖1 M PAFC‖Pt at 1 mV s^−1^. (g) Linear scanning voltammetry (LSV) curve of Pt‖1 M PAFC‖Pt scanned at 1 mV s^−1^. (h) Linear relationships between the oxidation and reduction peak current with the square root of the sweeping rate. (i) The charge and discharge profiles of cells in 1 M AlCl_3_, mixed 1 M AlCl_3_@FeCl_3_ and 1 M PAFC, respectively.

We first investigated the basic physical properties of three electrolytes, 1 M AlCl_3_, the mixed electrolyte of 1 M AlCl_3_ and 1 M FeCl_3_ (1 M AlCl_3_@1 M FeCl_3_), and 1 M PAFC, as shown in Fig. 2a. Obviously, the aluminum chloride solution changes from colourless to rust colour after the introduction of iron ions. The evident Tyndall effect in the PAFC electrolyte indicates the possible change of the status of aluminum ions and iron ions after the polymerization process. The obtained PAFC electrolyte was later freeze-dried, and the resultant powders were characterized through the thermogravimetric analysis (TGA). The results show that the contribution of the physical adsorption of water to the total weight of PAFC is ∼50%, but the contribution of coordination water and other chemical bond fractures to the total weight of PAFC is ∼13% as shown in Fig. S2.† Here, the freeze-dried powder of PAFC tested by X-Ray Diffraction (XRD, Fig. S3†) shows that the powder has a certain crystal structure, and scanning electron microscopy (SEM, Fig. S4a and b†) shows that the powder consists of large particles. As a result, the ion conductivity of PAFC is reduced to 50 mS cm^−1^ when compared to 1 M AlCl_3_ (110 mS cm^−1^) and 1 M AlCl_3_@1 M FeCl_3_ electrolyte (100 mS cm^−1^) as shown in Fig. 2b. To clarify this reduction, we carried out the Fourier transform infrared (FT-IR, Fig. S5a†) spectroscopy and Raman spectroscopy (Fig. S5b†) of PAFC. The newly generated peak belongs to hydroxyl irons (Fe–OH ,HO–Fe–OH or (HO)_2_–Fe–OH).^26^ In addition, Raman spectroscopy (Fig. S6†) was used to examine the three electrolytes and deionized water, revealing a noticeable distinctive peak (∼314 cm^−1^) following the addition of iron ions, and this also demonstrates that the polymerization event shifts the absorption peak of the iron functional group. To investigate the electrochemical properties of our PAFC electrolytes, we assembled a flooded cell using NiFe-PBA as the cathode and c-Al as the anode. Fig. S5† depicts the fundamental parameters of the NiFe-PBA cathode material.^27^ Powder XRD (Fig. S7a†) confirms that NiFe-PBA is compatible with those previously reported in the literature;^28,29^ both transmission electron microscopy (TEM) (Fig. S7b†) and SEM (Fig. S7d†) reveal the PBA cube's basic structural features and cubic shape with sizes of 100 nm and 200 nm, respectively. Thermogravimetric analysis (Fig. S8†) is also carried out to ensure that NiFe-PBA is compatible with previous reports.^28^Fig. 2c shows the cyclic voltammetry (CV) curve for NiFe-PBA‖1 M AlCl_3_‖c-Al with a scan rate of 1 mV s^−1^; there are two pairs of redox peaks, 1.1 V and 1.52 V *vs.* Al/Al^3+^, respectively, which are attributed to the insertion of aluminum ions in different sites in NiFe-PBA, as reported in the literature.^30^ Comparably, in Fig. 2d, the CV curve of NiFe-PBA‖1 M AlCl_3_@1 M FeCl_3_‖c-Al at the sweep rate of 1 mV s^−1^, the dominant reaction plateau is located at 1.25 V; it may involve the intercalation of Al in NiFe-PBA and the surface redox reaction of iron as described in eqn (1) and (2) with the output voltage of 1.14 V.

Cathode:

1 Fe^3+^ + e^−^ ↔ Fe^2+^, 0.7 V *vs.* SHE

Anode:

2 Fe^2+^ + 2e^−^ ↔ Fe, −0.44 V *vs.* SHE

Two reactions occur in parallel in the cell, with the main reactions overlapping each other. Fig. 2d shows CV curves of NiFe-PBA‖1 M PAFC‖c-Al at different scan rates (1, 3, 5 mV s^−1^); it is evident that the oxidation peak is at ∼1.35 V, which is consistent with the cell in the mixed electrolyte, the shifting of the peak occurs due to the change of concentration and pH of the electrolyte. In addition, the CV and linear sweep voltammetry (LSV) measurements on PAFC with a platinum sheet as the counter electrode and working electrode are shown in Fig. 2e and f, respectively.

The peak variations located at 0.8 V matched the cathode's reaction potential in eqn (2). It can be seen more clearly in the purple region of LSV that the peak at −0.45 V corresponds to the reduction potential of ferrous ions. The linear connection between peak currents and the square root of sweeping rate, as shown in Fig. 2h, when combined with Fig. 2g, indicates that the reaction in PAFC is a diffusion-controlled mechanism (|*b*| > 1). Using the Randles–Sevcik equation below, the diffusion coefficient was calculated to be about 2 × 10^−8^ cm^2^ s^−1^.

3 *I* = 26 900 × *n*^1.5^*AD*^0.5^*v*^0.5^*C*

where *I* is the peak current, *n* is the number of electrons involved, *A* is the active surface area, *D* is the diffusion coefficient, *v* is the sweeping rate, and *C* is the concentration of redox species. While the derived coefficient is not comparable with those of conventional redox species in water, the value is reasonable at such a high concentration. Furthermore, the comparison of the galvanostatic charge/discharge (GCD) in the three different electrolytes is shown in Fig. 2i. It is evident that the battery using the PAFC electrolyte possesses the largest specific volumetric discharge capacity of 35 A h L^−1^ compared to that of cells in 1 M AlCl_3_ (6 A h L^−1^) and 1 M AlCl_3_@1 M FeCl_3_ electrolyte (11 A h L^−1^).

**Fig. 3:**
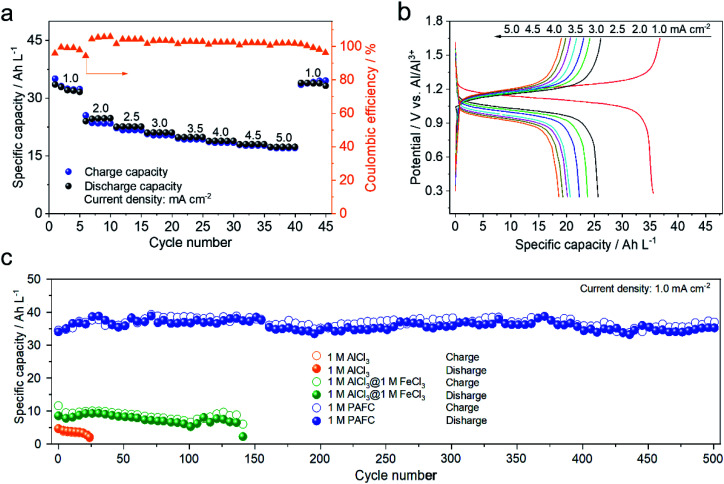
Evaluation of the electrochemical performances of the PAFC electrolyte based HIAAIB. (a) The rate performance and coulombic efficiency of NiFe-PBA‖1 M PAFC‖c-Al at various current densities. (b) The charge–discharge profiles of NiFe-PBA‖1 M PAFC‖c-Al for constant current at various current densities. (c) Under the same conditions, the capacity cycling performance of NiFe-PBA‖c-Al in three different electrolytes.

To further demonstrate the electrochemical performance of HIAAIB, we studied the rate capability and the cycling performance of the cell in detail. Fig. 3a depicts the rate capability of NiFe-PBA‖1 M PAFC‖c-Al. It is clear that the specific capacity gradually decreases with the increase of current densities. Impressively, the cell still delivers the specific volumetric capacity of 20 A h L^−1^ when the current density increases to 5.0 mA cm^−2^. The specific volumetric capacity is 37 A h L^−1^ when the current density sets back to 1.0 mA cm^−2^, with no obvious attenuation, indicating the excellent rate capability of the HIAAIB.

Meanwhile, Fig. 3b depicts the GCD curve of NiFe-PBA‖1 M PAFC‖c-Al at different current densities, verifying the stable and flat discharge plateaus with the increased current densities. The highest current density can approach 1 mA cm^−2^ in Fe(126) deep eutectic solvent (DES) systems according to the literature.^31,32^ Comparably, the capacity decrease is not visible in HIAAIB when the current density is between 2.5 and 5.0 mA cm^−2^, indicating that the battery with the PAFC electrolyte shows excellent rate performance. At the same time, the coulombic efficiency was maintained at around 90%, which is outstanding among the reported aqueous aluminum ion batteries. The enhanced coulombic efficiency should originate from the low electrochemical impedance of the cell (electrochemical impedance spectroscopy (EIS) comparison of the three different electrolytes, Fig. S9†) and restraining the anodic hydrogen evolution by the alloying process in 1 M PAFC. The excellent rate performance and high coulombic efficiency are attributed to the unique operation mechanism and chemical characteristics of the PAFC electrolyte. The cycling performances of batteries in the three different electrolytes under the same conditions are compared in Fig. 3c. The cycling performance and capacity retention rate are superior in the PAFC electrolyte. The initial discharge capacity is 6 A h L^−1^ in 1 M AlCl_3_, and the cycling shows severe decaying after 25 cycles. In the mixed electrolyte of 1 M AlCl_3_ and 1 M FeCl_3_, the discharge capacity increased to 11 A h L^−1^, but it suffered the decaying problem after 145 cycles. Impressively, the reversible discharged specific capacity is as high as 38 A h L^−1^ in 1 M PAFC electrolyte. Moreover, the specific capacity remains at 35 A h L^−1^ without obvious decaying after 500 cycles. Such high volumetric capacity originates from the surface redox reaction of ferric ions as demonstrated in the mechanism in Fig. 1. More importantly, the excellent cycling performance arises from the complex status of aluminum ions, and ferric ions reduced the number of free water molecules, thus hindering the hydrolysis reaction on the anode.^11,33^

**Fig. 4:**
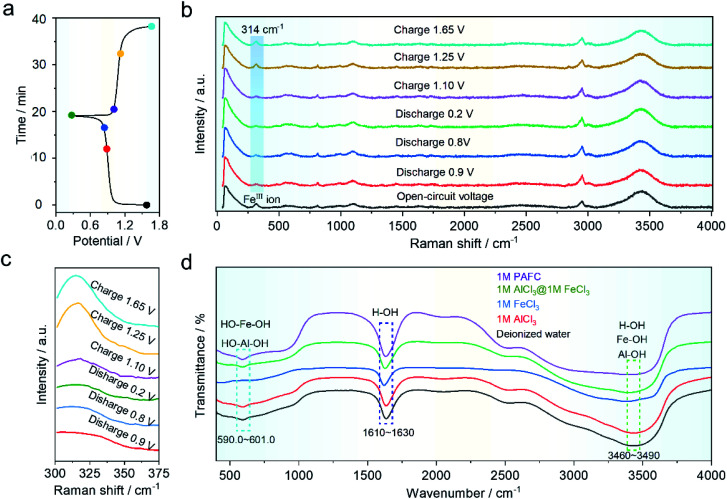
Investigation of PAFC electrolyte by *ex situ* electrochemical measurements at the different charge/discharge states. (a) Potential–time curve during a battery cycle for NiFe-PBA‖1 M PAFC‖c-Al. (b) Raman spectra of the electrolyte at various charge and discharge voltage positions. (c) Raman spectra of the blue magnified region. (d) PAFC and other electrolytes' FT-IR spectra are compared.

In order to understand the mechanism of the HIAAIB, we conducted *ex situ* Raman and FT-IR spectra of the electrolyte under various charging and discharging states, as shown in Fig. 4a. The samples are collected at different charging/discharging states as shown in the time–voltage curve (Fig. 4a). As shown in Fig. 4b, the Raman peak located at ∼314 cm^−1^ belongs to ferric ions;^29,34^ the blue gradient area in Fig. 4b has been expanded in Fig. 4c to make it easier to observe the change of peak value. The peak value decreased with the extent of the discharge of electrolyte from 0.9 to 0.2 V, indicating that ferric ions are being reduced to ferrous ions in the electrolyte, while during the charging process (from 1.10 to 1.65 V), the peak eventually becomes similar to that in the open-circuit voltage (OCV) state, indicating that the oxidation/reduction of iron ions is highly reversible in the charging and discharging process, which is also the major reason for the incensement of capacity in the HIAAIB. Furthermore, the *ex situ* FT-IR spectra of PAFC are shown in Fig. 4d. Aluminum ions and iron ions are polymerized to varied degrees in the figure with the addition of ferric chloride. The peak shift of H–OH in the range 1610–1630 cm^−1^ (cyan region) indicates that H–O is largely replaced by aluminum or iron ions, causing a peak shift.^35^ PAFC peaks in the 590–601 cm^−1^ (blue area) and 3460–3490 cm^−1^ (green area) ranges after polymerization are asymmetric chain structures corresponding to the synthesis of hydroxyl aluminum and hydroxyl iron.^36^ This unique structure has the ability to rapidly conduct non-hydroxyl ions.

**Fig. 5:**
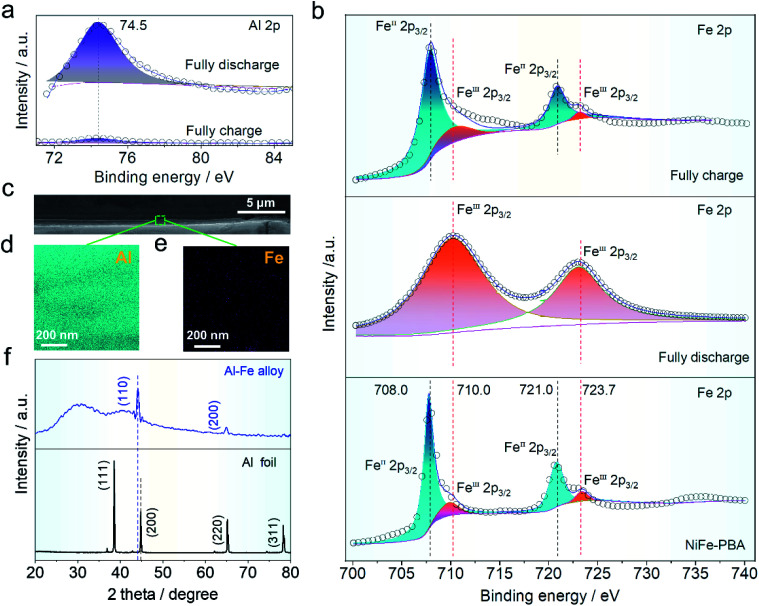
Characterization of the cathode and anode in the HIAAIB. (a) After etching, the Al 2p peak value of NiFe-PBA changed in full charge and full discharge states. (b) The peak value variations of Fe 2p in the original NiFe-PBA structure during fully charged and fully discharged states, respectively. (c) Cross-sectional SEM images of the aluminum anode after cycling. (d and e) The EDS mapping of Al and Fe at the cross-section of the aluminum anode after cycling, respectively. (f) Comparison of the aluminum anode before and after cycling by *ex situ* XRD.

X-ray photoelectron spectroscopy (XPS) was used to characterize the valence states of aluminum and iron in the cathode material. The binding energy in aluminum concentration (∼74.5 eV) corresponding to Fig. 5a before and after the charge and discharge can be readily seen, indicating that aluminum ions in various charge/discharge states exhibit reversible inlay behavior. The intensity distribution of aluminum ions before and after charging and discharging of NiFe-PBA is intuitively depicted in Fig. S8a and b,† indicating that aluminum ions are intercalated into the host structure after discharging. At the same time, the iron distribution on the surface was tested under the charge and discharge states. Intensity distribution changes slightly and is not obvious, which may be due to the iron from the NiFe-PBA (Fig. S10c and d†). Fig. 5b shows the fine-scanned Fe 2p spectra, which were fitted to investigate the change of valence states. In Fig. 5b, the binding energy of Fe 2p of NiFe-PBA at 708.0 and 721.0 eV corresponds to Feii valence state, this peak shifted to higher binding energies of 710 and 723.7 eV (Feiii) when fully discharged due to the oxidation of Feii. But the valence state of iron ions recovers to that of NiFe-PBA when fully charged again, indicating the reduction of Feii by the intercalation of aluminum ions. In addition, the cathode electrode was examined by SEM (Fig. S11a and b†) and TEM (Fig. S12a and b†) after discharge, indicating that the cubic structure of PBA did not change after discharge. The operation mechanism of the HIAAIB can be further elucidated on the anode. Here, in order to further explain the reversible deposition/stripping process of iron on the cathode surface, a three electrode system (NiFe-PBA as the working electrode, AlN + graphite as the counter electrode, saturated calomel as the reference electrode, in which both the working electrode and the counter electrode use carbon cloth as the collector) is set up.1 M PAFC is used as the electrolyte for testing, which can realize a long cycle (2000 cycles) at 1.0 mA cm^−2^ current density, and further explains the reversible reaction of iron on the cathode surface (Fig. S13†).

The SEM comparative findings following the reaction are shown in Fig. 5c; to further confirm the alloying process, the energy dispersive spectroscopy (EDS) division maps of aluminum and iron in the green region are presented in Fig. 5d and e. The coexistence of Fe and Al elements in the deposition layer suggests the generation of the Fe–Al alloy.^37–39^ The *ex situ* XRD comparison of the main components on the anode surface before and after operation is shown in Fig. 5f. Clearly, the peak strength of aluminum in the anode decreased after cycling when compared to the pure aluminum anode, while the highest deviation occurs at 45°, showing that new substances are formed after the operation.^3,40,41^ The peak located at 44.2° is the apex of Al–Fe, indicating that the alloy reaction has occurred.^42,43^ Furthermore, digital photos of the aluminum anode were taken before (Fig. S14a†) and after (Fig. S14b†) the operation. It was found that the aluminum anode after the reaction closely adhered to the magnet, which helped to explain the occurrence of the aluminum anode alloy reaction (Fig. S14c†).

## Conclusions

To summarize, we demonstrated a high-performance hybrid-ion battery using a strongly hydrolyzed/polymerized aluminum–iron hybrid electrolyte. The obtained energy density is as high as ∼42 W h L^−1^ at a specific volumetric capacity of 35 A h L^−1^, and the specific volumetric capacity remains at 20 A h L^−1^ at a high current density of 5 mA cm^−2^. These outstanding performances are attributed to an inorganic polymer hybrid electrolyte derived from aluminum and iron polymerization, which triggers the intercalation of aluminum ions in NiFe-PBA and the reduction of ferric ions on its surface in parallel. Furthermore, the Fe–Al alloy generated at the anode promotes the deposition of aluminum ions, improving the cycling performance of the battery. Our work paves the way to developing high-energy batteries through the rational design of hybrid-ion electrolytes.

## Experimental

### Cathode preparation

A simple precipitation process was used to manufacture NiFe-PBA. In detail, 0.5 mmol K_3_[Fe(CN)_6_] was first dissolved in 20 mL of water, followed by dropping a solution of 0.5 mmol nickel chloride (NiCl_2_·6H_2_O) and 1 mmol sodium citrate in another 20 mL of water. The precipitates were generated after 24 hours of stirring in a round-bottom flask under magnetic stirring at room temperature. The precipitates were centrifuged and carefully cleaned with deionized water multiple times after another 24 hours of aging. To get the final NiFe-PBA samples, the precipitates were dried at 80 °C under vacuum. In this process, the cathode materials (NiFe-PBA powder) were mixed with Ketjen black (KB) and polyvinylidene fluoride (mass ratio: 7 : 2 : 1) in *N*-methylpyrrolidinone (NMP) to obtain a mixed slurry. Then, the slurry was coated onto carbon cloth (Shanghai Hesen Electric Co., Ltd, China) and dried at 120 °C in a vacuum oven for 12 hours.

### Anode preparation

To make the mixed powder, industrial nano AlN powder and industrial graphite powder were proportioned at a mass ratio of 7 : 3 and thoroughly pulverized in a mortar. The powders were calcined in an air environment at 650 °C for 3 hours at a rate of 5 °C min^−1^. To make an electrospray slurry, the calcined mixed powder was combined with PVDF in an 8 : 2 ratio and pulverized. The produced paste (composed of AlN, graphite and NMP) was electrosprayed onto a polished metal aluminum foil at 24 kV and with the rate of 3 mL h^−1^. The aluminum foil after electrospraying was dried in an oven at 80 °C for 12 hours.

### Electrolyte preparation

0.033 M anhydrous FeCl_3_ and 0.3 M AlCl_3_·6H_2_O (*n* (Fe) : *n* (Al) = 1 : 9) were dissolved in 5 mL and 12 mL deionized water, respectively. FeCl_3_ solution was added to AlCl_3_ solution and mixed at 80 °C. Then 3 mL NaOH (2.5 M) was dropped into the combined solution at the rate of 2 drops per minute. The mixture was sealed, heated, and stirred for 2 hours. And the concentration of PAFC was calculated based on the concentration of AlCl_3_. The volume of the electrolyte is 10 mL and is applied for the capacity calculation.

### Assembly and electrochemical measurements of the battery

All electrochemical cells were made with two electrodes in an electrolytic system. A Neware BTS-53 system was used to perform galvanostatic charge/discharge cycling at varied current rates and a voltage range of 0.2–1.65 V (*vs.* Al/Al^3+^). The CV curves were recorded using an electrochemical workstation (CHI660E) at a scan rate of 1 or 5 mV s^−1^. Electrochemical impedance spectroscopy (EIS) was done in the frequency range of 0.01 Hz–100 kHz with a 10 mV amplitude using an auto-lab electrochemical workstation. All electrochemical testing was performed at room temperature.

## Data availability

All experimental data, and detailed experimental procedures are available in the ESI.†

## Author contributions

Renqian Tao: conceptualization/data curation/formal analysis/investigation/writing – original draft/writing – review & editing/visualization. Caitian Gao: resources/software/supervision. Erqing Xie: project administration/validation. Bin Wang: funding acquisition. Bingan Lu: funding acquisition/methodology.

## Conflicts of interest

The authors declare no conflict of interest.

## Supplementary Material

SC-013-D2SC03455G-s001
