# Supplementary material for: A stable and high-energy aqueous aluminum based battery
Source: Chem Sci. 2022 Jul 28;13(34):10066–73. doi: 10.1039/d2sc03455g (PMC9430682; doi:10.1039/d2sc03455g)
Supplement: SC-013-D2SC03455G-s001 [file SC-013-D2SC03455G-s001.pdf]

Electronic Supplementary Material (ESI) for Chemical Science.
This journal is © The Royal Society of Chemistry 2022

Supporting Information

A stable and high-energy aqueous aluminum based battery

Renqian Tao[a], Caitian Gao[b,], Erqing Xie[a,*], Bin Wang[c], and Bingan Lu[b]*

[a] *Key Laboratory for Magnetism and Magnetic Materials of the Ministry of Education, School of Physical Science and Technology, Lanzhou University, Lanzhou 730000, P. R. China*

[b] *School of Physics and Electronics, State Key Laboratory of Advanced Design and Manufacturing for Vehicle Body, Hunan University, Changsha 410082, P. R. China*

[c] *School of Physics and Electronic Engineering, Xinxiang University, Xinxiang 453000, P. R. China*

Materials Characterizations:

The samples were characterized by Fourier transform infrared (FT-IR, Thermo Scientific Nicolet iS20) spectroscopy. Thermogravimetric (TG) was used to explore the bonding state and the moisture content of PAFC. The field emission scanning electron microscope (FESEM, TESCAN MIRA3 GMH) and transmission electron microscopy (TEM, Titan G2 60-300 with image corrector) were adopted to characterize the morphology. The elemental distributions were determined by the energy dispersive spectrometer (EDS) mapping analyses, and the surface elemental valence states were evaluated by an X-ray photoelectron spectrometer (XPS, ESCALAB 250Xi) using the 200 W Al Kα radiation. The X-ray diffraction (XRD) result was collected using Cu Kα1 radiation in the range of 2θ = 5-80° with the scanning speed of 10° min^{-1}.

XPS characterization of cathode material. After operation, the carbon cloth covered with cathode material is vacuum dried for 12 hours. Before the test, the sample was etched by 10 nm for two times, avoiding the surface contamination from the electrolyte.

Raman analysis of electrolyte. The sample test should be performed once the battery is stable (approximately 20 cycles). The sample point must be set at the same position, and the amount must be kept to a minimum of 2 mL each time. The test must be performed as soon as the sample is done.

SEM characterization of anode material. After 12 hours vacuum drying period, the aluminum negative electrode material should be removed, packaged, and kept for SEM testing.

Electrochemical measurements. The average mass loading of active material on the cathode is 1.5-2.0 mg cm^{-2}. The charge/discharge tests were performed using the Neware BTS-53 system in the voltage range of 0.2-1.65 V. The CV curves were recorded using the electrochemical workstation (CHI660E) at a different scan rates. All electrochemical testing was performed at room temperature.

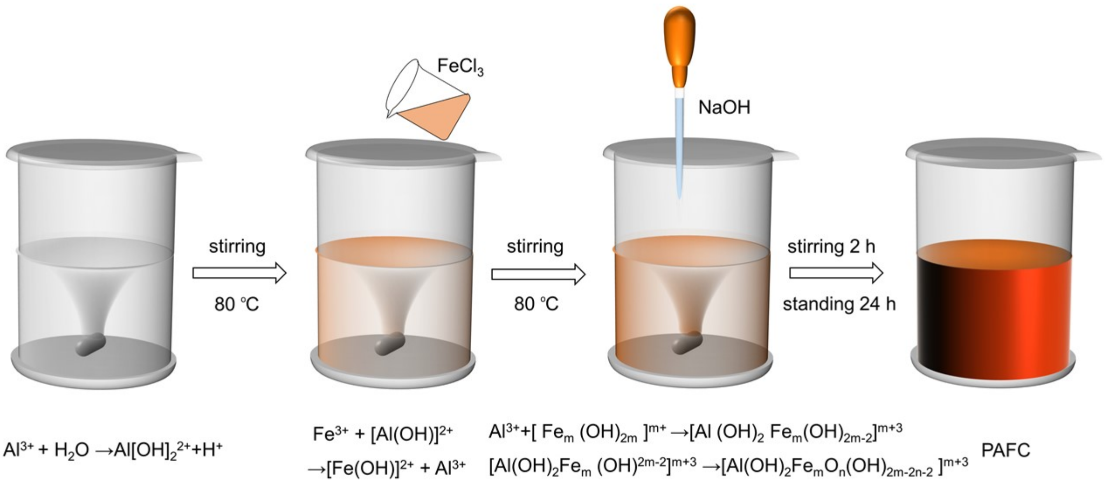

Figure S1. Schematic diagram of preparation process of PAFC electrolyte.

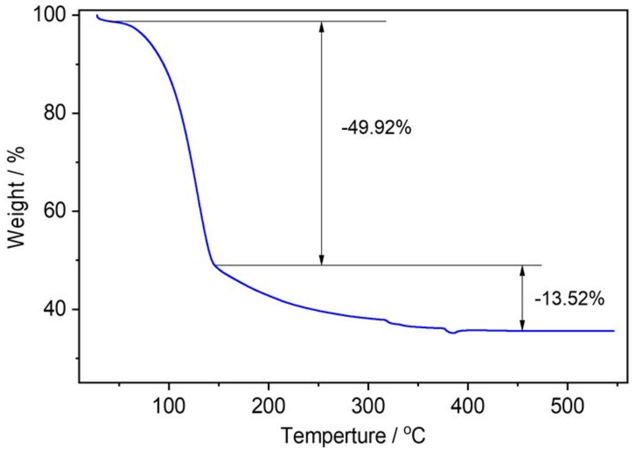

Figure S2. The TGA of PAFC powder after freeze-drying.

When the temperature of freeze-dried PAFC powder is changed from 50°C to 140°C, the water evaporates rapidly, as shown in Fig. S2. At this time, the proportion of physical adsorption water to the total weight of PAFC is ~50%. As the temperature rises, the water evaporates, but at a much slower rate, until the temperature reaches 450°C and the weight of the water remains the same. Mainly due to the volatilization loss of coordination water and other chemical bond fracture, only accounted for the total proportion of ~13%.[1-3]

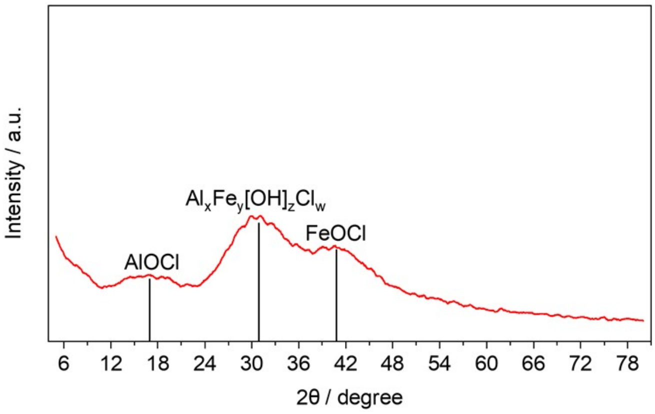

Figure S3. The XRD results of PAFC powder show that it has a certain crystal structure

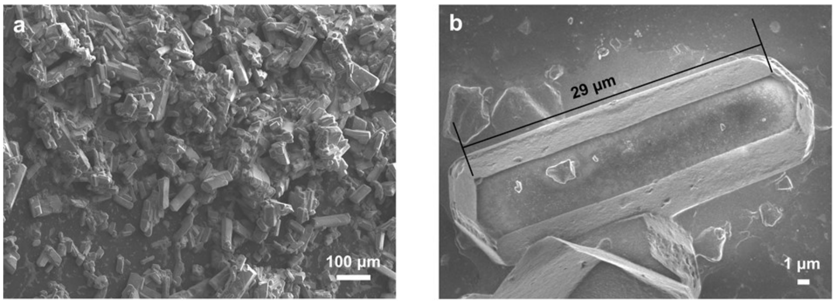

Figure S4. The SEM results of PAFC powder showed that it had the morphology of large particle with the size of 29 μm.

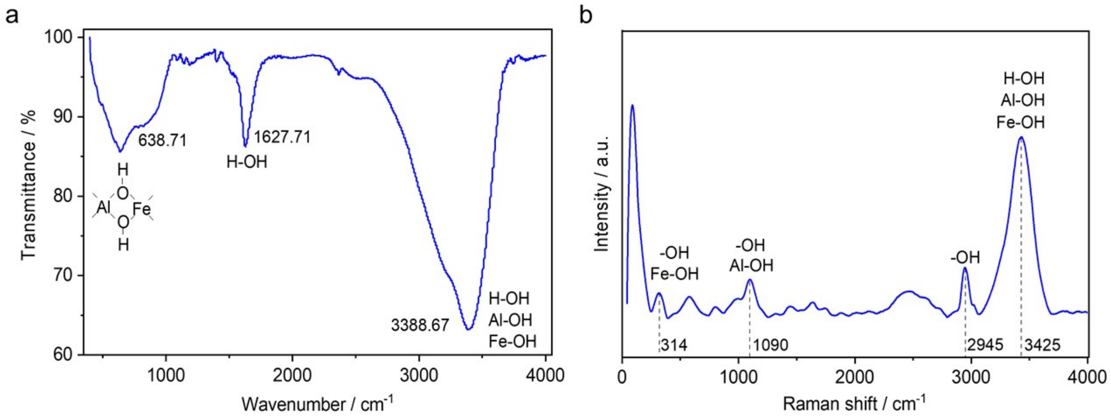

Figure S5. a) The Fourier transform infrared (FTIR) spectroscopy of PAFC. b) Raman spectra test of PAFC.[4, 5]

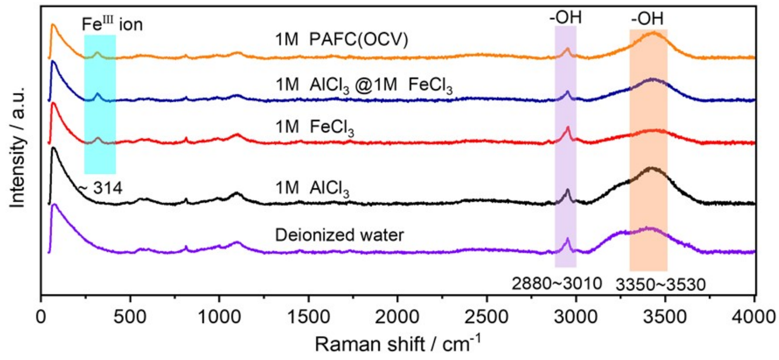

Figure S6. The comparison of Raman spectra of deionized water and four electrolytes.

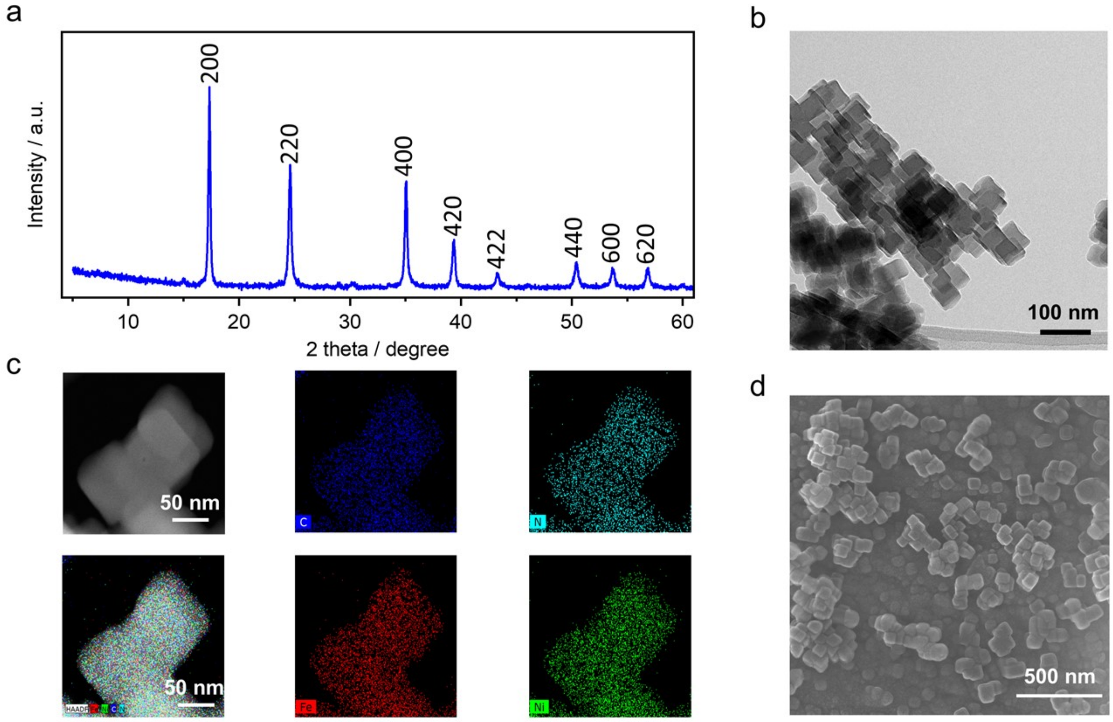

Figure S7. The basic properties of NiFe-PBA, a) XRD spectrum, b) TEM image, c) TEM and TEM mapping, d) SEM image.

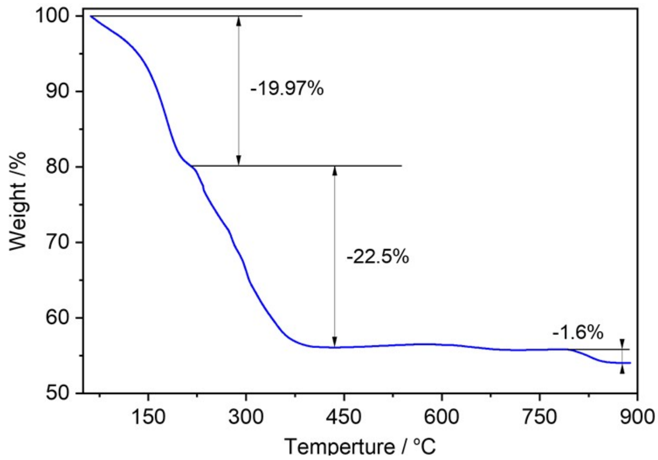

Figure S8. Thermogravimetric analysis of NiFe-PBA

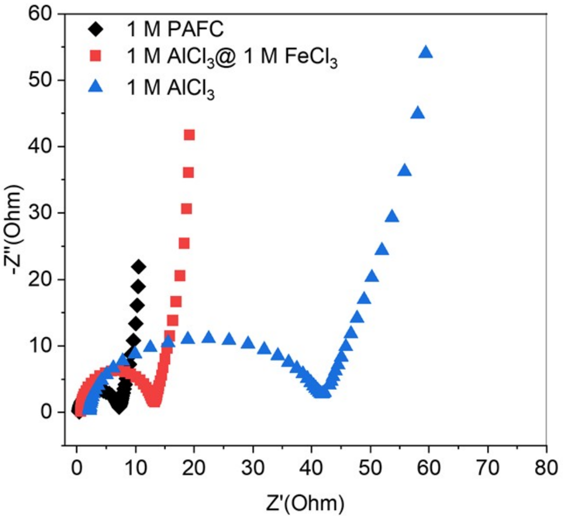

Figure S9. Electrochemical impedance spectroscopy of three different electrolytes (1 M PAFC, 1 M $AlCl_3$@1 M $FeCl_3$ and 1 M $AlCl_3$) under the same cathode (NiFe-PBA) and anode (c-Al).

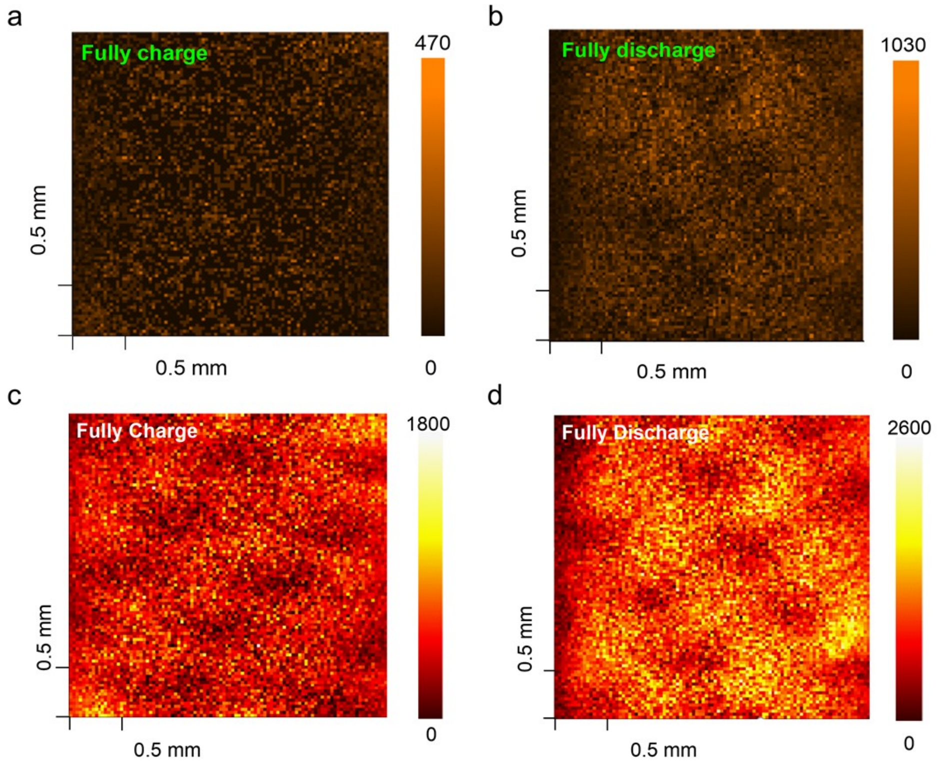

Figure S10. The intensity distribution of aluminum ions (a and b) and iron ions (c and d) before and after charging and discharging of NiFe-PBA

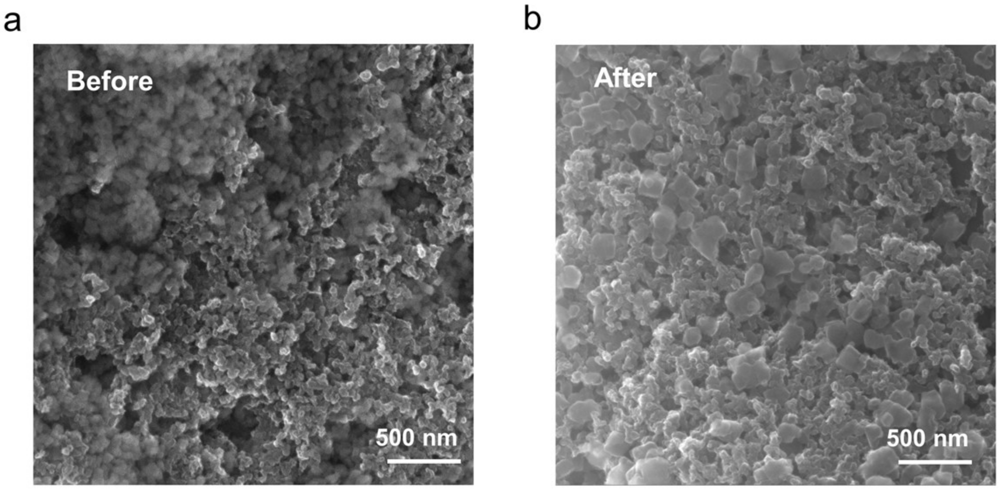

Figure S11 SEM image of NiFe-PBA coated carbon cloth before and after discharge

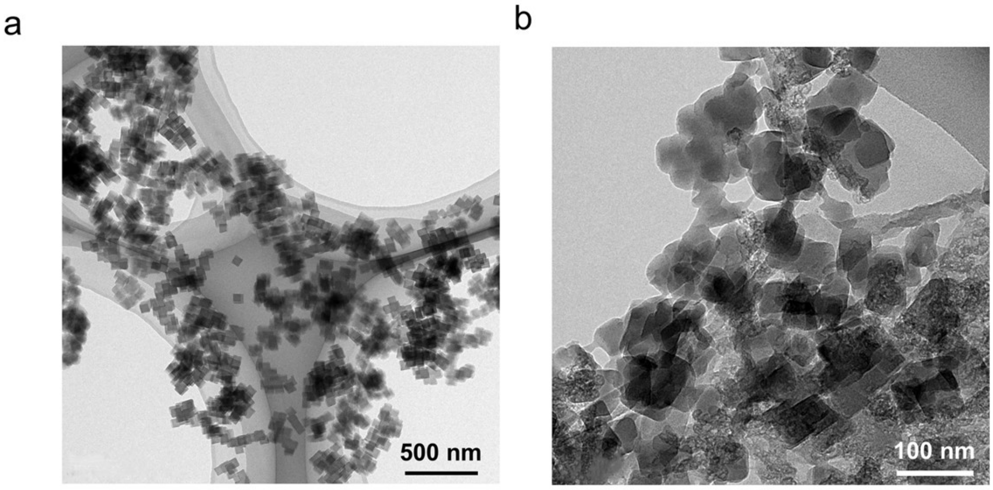

Figure S12 TEM image of NiFe-PBA coated carbon cloth after discharge.

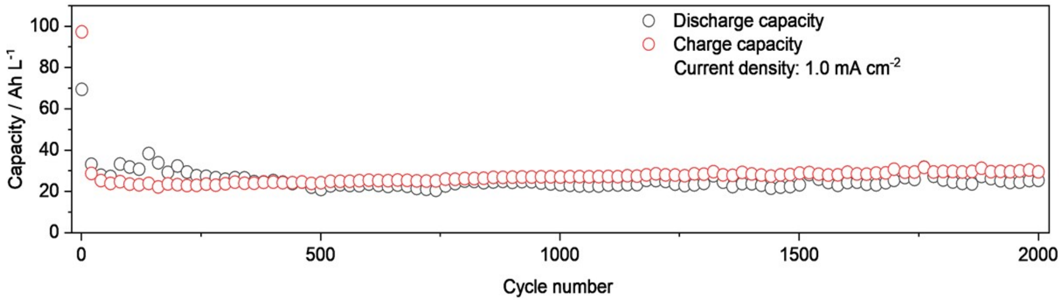

Figure S13. Three electrodes at current density of 1.0 mA cm^{-2} (NiFe PBA as working electrode, AlN+ graphite as counter electrode, saturated calomel as reference electrode), cycle performance and discharge capacity.

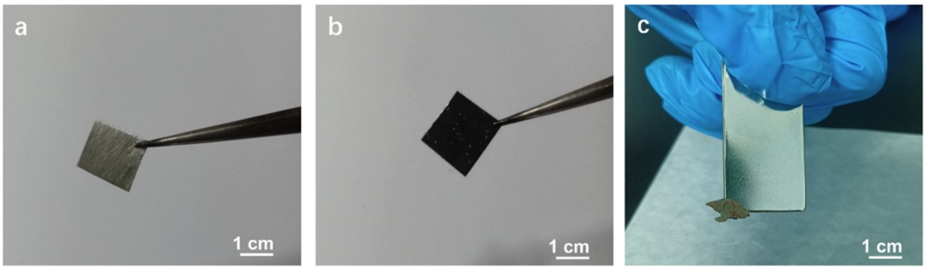

Figure S14. Aluminum anode before electric fuel injection (EFI) (a), after EFI (b) and after operation(c), and the anode after operation has strong magnetism

.

Reference

1. Y. Sun, J. Liu, W. Sun, H. Zheng and K. J. Shah, *Desalination and Water Treatment*, 2019, **167**, 13-26.
2. X. Ren, X. Hu, W. Cheng, S. Bian, Y. Zhao, M. Wu, D. Xue, Y. Li, W. Lu and P. Wang, *Fuel*, 2020, **267**, 117261.
3. K. E. Lee, N. Morad, T. T. Teng and B. T. Poh, *Chemical Engineering Journal*, 2012, **203**, 370-386.
4. Y. Wang, W. Sun, L. Ding, W. Liu, L. Tian, Y. Zhao, M. Zhang and X. Wang, *Journal of Water Process Engineering*, 2021, **40**, 101847.
5. X. Liu, B. Wang, D. Wang, J. Cheng, Q. Meng, Z. Zhang, P. Gao, J. An, J. Lou and M. Li, *Fuel Processing Technology*, 2019, **193**, 372-377.
